# Politico-Epistemic Tensions Regarding Personal Assistance and Care for People with Disabilities: An Integrative Literature Review

**DOI:** 10.3390/ijerph20021366

**Published:** 2023-01-12

**Authors:** Juan Andrés Pino-Morán, Pía Rodríguez-Garrido, María Soledad Burrone

**Affiliations:** 1 Instituto de Ciencias de la Salud, Universidad de O’Higgins, Rancagua 2820000, Chile; 2 Millennium Nucleus Studies on Disability and Citizenship (DISCA), Rancagua 2820000, Chile; 3 Grupo de estudios críticos de la discapacidad (CLACSO), Buenos Aires C1101AAX, Argentina; 4 Women, Health and Ethics Study Group, University of Barcelona, 08907 Barcelona, Spain; 5 Laboratório de Estudos Sociais sobre o Nascimento, nascer.pt, Instituto Universitario de Lisboa, 1649-026 Lisboa, Portugal

**Keywords:** care, disability, ethic of care, feminism of disability, personal assistance

## Abstract

Background: Since the 1960s, the Independent Life Movement has demanded personal assistance as a right for people with disabilities to access autonomy. In turn, feminist movements have shown a special concern for the care and profile of the providers. Both postures have created tensions around the provision of personal assistance and care for people with disabilities. Aim: To know and analyze the scientific evidence regarding approaches to personal assistance and care for people with disabilities. Methods: An Integrative Literature Review using five databases: Dialnet, Scielo, PubMed, Scopus, and Web of Science. The Boolean combinations were: “Personal assistance AND disability”; “Personal assistance AND care AND disability”; “Care AND disability” in English, and “Asistencia personal AND discapacidad”; “Asistencia personal AND cuidados AND discapacidad”; “Cuidados AND discapacidad” in Spanish. A total of 31 scientific articles were obtained. A content analysis was then, with five analysis dimensions emerging. Results: The articles approached the positive aspects of personal assistance. Others established the need for more resources in order to not be an exclusive reality for developed countries. Profiles were made of racialized, young, migrant women as the identity behind (informal) care. From the perspective of a feminist disability care ethic, new forms of providing care are proposed, by changing the focus from individual and family responsibility, towards a social and collective focus. Conclusion: The evidence analyzed considers various dimensions of the epistemo-political tension between personal assistance and care. The meeting point between both perspectives is interdependence and autonomy; on the one side, for people with disabilities, and on the other, for the women profiled as the main caregivers.

## 1. Introduction

Personal assistance (PA) arose during the 1960s with the figure of Edward Verne Roberts (1930–1995) a major activist in the Independent Living Movement in the USA. Based on his experience as a student with a disability, he left clear evidence about the vicissitudes of universal access within the university [1].

PA rose from the processes of de-institutionalization and de-familization. Both processes fit within a biomedical, conservative, and assistentialist logic regarding people with disabilities. The de-familization process involves moving the focus of mandatory care for people with disabilities beyond the intimate or family environment. In turn, the de-institutionalization process involves changing the pathological perspective on disability and bodies beyond the “sole solution” of the demands of medicine and institutions. In both scenarios, the figure of PA “supports independent life, allowing them to carry out everyday life activities without the constant participation of a friend, family member, volunteer or solidarity from strangers” [2] (p. 73).

The Convention on the Rights of Persons with Disabilities, 2006, established the right to live independently and to be included, mentioning PA within it:

“Party States to this Convention recognize the equal right of all persons with disabilities to live in the community, with choices equal to others, and shall take effective and appropriate measures to facilitate full enjoyment by persons with disabilities of this right and their full inclusion and participation in the community, including by ensuring that: (a) Persons with disabilities have the opportunity to choose their place of residence and where and with whom they live on an equal basis with others and are not obliged to live in a particular living arrangement; (b) Persons with disabilities have access to a range of in-home, residential and other community support services, including personal assistance necessary to support living and inclusion in the community, and to prevent isolation or segregation from the community; (c) Community services and facilities for the general population are available on an equal basis to persons with disabilities and are responsive to their needs” [3] (p. 15).

Various European countries have ratified the Convention and the figure of the PA. In various cases, funding for personal assistants has been via direct payment from people with disabilities, via independent living offices which are mediated between both figures, and via an external entity. However, the “union” between the personal assistant and the disabled person (or their legal representative) is via a job contract [4].

From the perspective of disability, care connotes an uncomfortably obligatory situation involving oppression and a loss of autonomy, particularly when conducted by a family member [5]. In this sense, the figure of the caregiver in the disability area is usually reflected in a direct family member or a close friend who provides unpaid care [6]. From this logic, care moves away from the capacity for choice that PA provides for people with disabilities. In turn, and according to the World Bank [7], this is more often expressed in poor or developing countries.

From the perspective of feminist movements, care has been the focus of important demands, with the core purpose of moving its performance from the private/domestic sphere, where it has historically been given informally and without payment by women (mostly), out into the public sphere. This perspective “shift” involves understanding care from a political perspective where citizenship takes on a social commitment, and decision-makers establish paid formal care systems for their practitioners [8].

In line with this, the politico-epistemic tension between personal assistance and care includes various elements which deserve to be approached. This is particularly because of the current rise in critical disability studies, and the emergency that feminist movements and studies manifest in the face of concerns over caregivers’ profiles and conditions.

Information about the epistemo-political tension between personal assistance and care is still incipient; however, we can see a fertile field for future articulations. For this reason, our aim was to know and analyze the scientific evidence regarding approaches to personal assistance and care for people with disabilities (This study took place as part of a 2022 Fondecyt Postdoctoral Project from the National Research and Development Agency [ANID] titled “Socio-community care for people with disabilities in the O’Higgins region” (Folio n°: 3220665). The present article represents the development of the first research objective).

## 2. Material and Methods

The methodological framework which guided this study was an Integrative Literature Review. According to Goris [9] the objective of this type of review is to “demonstrate that the author has broadly studied the literature and critically evaluated its quality. It goes beyond merely describing the articles evaluated and includes a degree of analysis and conceptual innovation” [9] (p. 7). The choice of this methodology is appropriate due to its critical nature, which makes it possible to analyze the scientific evidence with greater emphasis. Whittemore [10], in turn, indicated the existence of five stages to apply an Integrative Literature Review.

### 2.1. Problem Identification

Since the 1960s, the Independent Living Movement has demanded personal assistance as a right for people with disabilities to be able to access more autonomy and independence. In turn, feminist movements have shown particular concern for the care and caregivers’ profiles, since the latter are usually racialized, young migrant women, who frequently carry out this role informally and often without pay. According to this, both postures have shown tension about the provision of personal assistance and care. For this reason, our aim was to know and analyze the scientific evidence regarding approaches to personal assistance and care for people with disabilities.

### 2.2. Literature Search

The search strategy included three moments: (a) Finding the keywords which oriented the initial search. For this, we used the terms “care”, “disability” and “personal assistance”. The Boolean combinations used were: “personal assistance AND disability”, “personal assistance AND care AND disability”, “care AND disability” in English, along with “asistencia personal AND discapacidad”, “asistencia personal AND cuidados AND discapacidad”, “cuidados AND discapacidad” in Spanish. (b) The second moment involved searching the databases to carry out the search, namely: Dialnet, Scielo, PubMed, Scopus, and Web of Science (WoS). (c) Finally, we selected scientific articles which met the following inclusion criteria: written in English, Portuguese or Spanish, published in the last 5 years (2018–2022), open access, and applied to humans. Finally, we eliminated articles that were repeated and where reviewing the abstract showed that they did not meet the study objective.

### 2.3. Data Evaluation

Information evaluation was considered via the experience and academic trajectory of specialist researchers in the matter (the authors of this study). In turn, methodological rigor was guaranteed via two methods: (a) Information systematization according to the five stages proposed by Whittemore & Knalf [11]; (b) The Standards for Reporting Qualitative Research (SRQR) guide [12].

**Figure 1:**
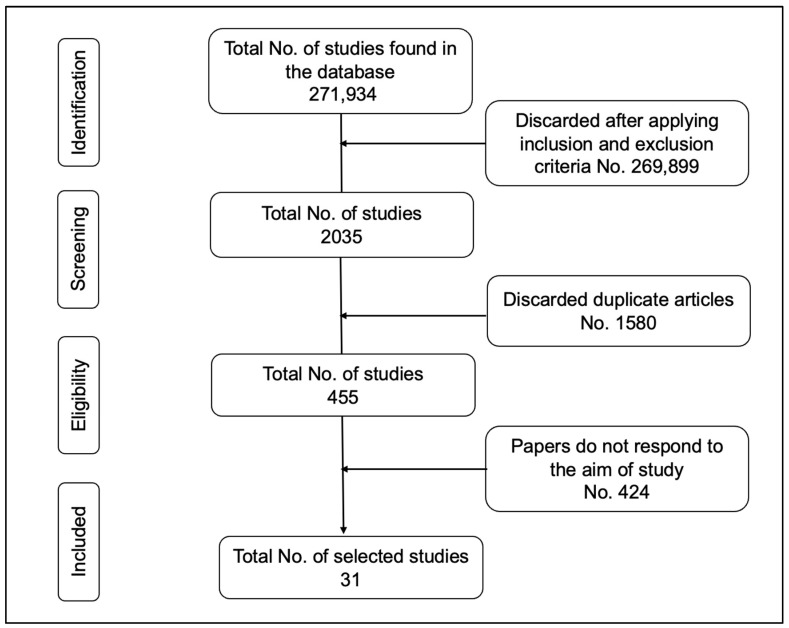
PRISMA flow chart for the literature search results. Source: Authors.

This gave us a total of 271,934 scientific articles (see Table 1). After applying the inclusion criteria, 31 scientific articles were selected (see Figure 1).

### 2.4. Data Analysis

After an initial reading, a descriptive analysis of the selected articles was conducted. In order to respond to the study objective, the next step was to analyze the content, which helped us go into depth in the theoretical-conceptual approach and development proposed by the selected articles. This information was available in five analysis dimensions: (1) Personal assistance: a tool for people with disabilities to achieve autonomy; (2) The familization of care versus personal assistance for people with disabilities; (3) Contractual vulnerability in care and personal assistance practices; (4) Ethics of care for people with disabilities; (5) Tension between the Independent Living Movement and the Feminist Movement over personal assistance and care.

### 2.5. Results (See Table 2)

The information is presented below, according to the database, journal, year, Boolean combination, authors, analysis dimension, title of the article, aims, methodological design, and conclusions. 

**Table 2:** Results.

Database	Journal	Year	Boolean Combination	Authors	Analysis Dimension (Results and Discussion)	Article	Aim	Design	Conclusion
Dialnet	Social Inclusion	2018	Personal assistance AND disability	Dietmar Rauch, Elisabeth Olin and Anna Dunér	2	A Refamiliarized System? An Analysis of Recent Developments of Personal Assistance in Sweden	To critically discuss in what direction Swedish disability support in the form of PA has been heading in the past decade when it comes to the balance of support responsibility between the public sector and the family	Theory Discussion	Data from the past decade about approval rates for AA-seekers as well as analyses of changed admission criteria suggest that we might be in the wake of a reversed development. Potential newcomers to the PA-system meet drastically decreased chances to pass the admission tests. Those already covered by PA run a heightened risk to lose their PA when their assistance needs are scrutinized in their next re-assessment.
Dialnet	Lus fugit: Interdisciplinary Legal-historical Journal	2019	Personal assistance AND disability	Rafael de Asís	1	Support and Personal Assistance	Exposing the legal construction of support and assistance provided to people in the framework of disability	Theoretical and conceptual reflection	Personal assistance training is needed, both to guarantee satisfying this right for people with disabilities and to protect assistants.
Dialnet	Spanish Disability Journal	2019	Personal assistance AND disability	Juan María Prieto Lobato, Pablo de la Rosa Gimeno, y José Luis Izquieta Etulain.	1	Personal assistance and mental disability: a service for social inclusion	Analyze one of the few experiences within the national territory of implanting a service for people with mental disabilities and/or severe mental illness	Qualitative study with interviews and discussion groups	Personal assistance represents a new way to help people with mental disabilities and severe mental illness. This service involves an authentic subversion of the traditional way of understanding and acting on the process of caring for this collective.
Dialnet	Journal of Political and Sociological Research (RIPS)	2020	Personal assistance AND disability	Mercedes López Pérez y Susana Ruiz Seisdedos	1	From the Independent Living Movement to personal assistance: the rights of people with functional diversity	Performing a theoretical study on the first social movement led by people with functional diversity themselves	Theoretical reflection	With the birth and rise of the ILM, people with functional diversity became aware of their rights. One of these is the right to live independently and with equal opportunities as other citizens. To facilitate this, personal assistance is indispensable.
Dialnet	Spanish Disability Journal	2018	Personal assistance AND disability	Antonio Iáñez Domínguez, José Aranda Chaves, y Julia García Romero.	1	Economic and social impact of personal assistance via Social Return on Investment methodology	Measuring the socioeconomic impact of a Project from the Independent Life Association of Andalusia.	Methodology applying Social Return on Investment	Research has shown the changes generated by the personal assistance Project for each agent of interest. All changes were quantified by SROI methodology, obtaining an approximation of the monetary value created by the personal assistance service.
Dialnet	TS Global–Social Intervention Studies	2020	Personal assistance AND disability	Mercedes López-Pérez y Carmen Álvarez-Nieto.	3	Personal Assistance in Spain. Perspectives from its protagonists: beneficiaries, people in charge of personal assistance at providing entities, and personal assistants.	Discovering and analyzing the current situation of this resource in Spain.	Mixed Study: Observational descriptive cross-sectional study and a qualitative study, via a semi-structured interview	There is little information about personal assistance, limited access to this resource, and job precariousness for personal assistance professionals.
Dialnet	Siglo Cero	2018	Personal assistance AND disability	Sandra Ruiz Ambit, Pablo Rodríguez Herrero, y Dolores Ixuxquiza Gasset	3	Personal assistants in the promotion of independent living for persons with intellectual disability: a basic and applied investigation	Identifying perceptions on support needs for independent living.	Mixed design. Descriptive cross-sectional quantitative design and case of study design.	This investigation can contribute to the field by opening new lines of research on the figure of the personal assistant, such as their own perception of the assistance process, the validation of training programmes following the competency profile outlined above, or an analysis of personal assistants’ support with multiple disabilities and serious communication difficulties.
Dialnet	Social Inclusion	2018	Personal assistance AND disability	Christoph Tschanz	3	Theorising Disability Care (Non-) Personalisation in European Countries: Comparing Personal Assistance Schemes in Switzerland, Germany, Sweden, and the United Kingdom	Examines four European countries regarding their degree of disability care personalization	Theoretical analysis	That conservative-corporatist welfare regimes provide less-supportive opportunity structures for policy change pertaining to personal assistance than other welfare regimes.
Dialnet	Siglo Cero	2020	Asistencia personal AND discapacidad	Alberto Minoletti, Pamela Gutiérrez, M° José Poblete, Bernardita López, Juan Bustos, Carla Muñoz, y Esteban Encina	1	Design of a brief personal assistance model for people with intellectual disabilities in Chile	Developing a personal assistance model for, initially, people with light and moderate intellectual disabilities.	Literature review and expert analysis (professionals, users, experience)	The proposed personal assistance model has the strength of being based on both evidence and experiences described in recent literature, along with expert opinions. It also has the advantage of gathering Latin American cultural traditions. One major weakness is the lack of national and international studies on the topic.
Dialnet	Civil Law Journal	2020	Care AND disability	Antonio Pau	5	The equality principle and the care principle, with special attention to disability	Comparing the legal and administrative implications of the equality and care principles	Theoretical review	No dividing line can be traced between people who need care and people who do not need care. In the public sphere, applying the care ethic must lead to attentive administrative action which is solicitous to citizens.
Dialnet	Contextos Journal	2021	Care AND disability	Diego Carmona Gallego	4	Autonomy in disability from the perspective of care ethics	Reflection on autonomy in the disability field, regarding the possibility of making decisions contemplating the diverse situations that this process can imply.	Theoretical review	Considering the fundamental importance of autonomy, we appeal to the configuration of this concept from a perspective that decouples it from the idea of self-sufficiency. This conceptual innovation arising from the contributions of complex thought and feminist philosophy has been shown to be more effective to describe the concrete experiences wherein decisions by people with and without disabilities take place, along with a lack of affiliation with the current idea centered on a self-sufficient individual.
PubMed	Sociology of Health & Illness	2020	Personal assistance AND disability	Tom Porter, Tom Shakespeare, and Andrea Stöckl	3	Performance management: a qualitative study of relational boundaries in personal assistance	To gain a deeper understanding of PA relationships, and to explore how both parties manage interpersonal challenges.	Qualitative design with interviews.	Personal assistance is a unique social relationship, which subverts typical interpersonal boundaries. Disabled employers and PAs often hold divergent views and preferences concerning the status of their relationships.
PubMed	International Journal of Environmental Research and Public Health	2022	Personal assistance AND disability	Ulrika J. Berggren and Ann-Sofie Bergman	2	Whether Disabled Parents Receive Personal Assistance for Parenting and the Consequences for Children–An Interview Study	To shed light on the meaning of PA for parents and children in everyday life, especially when PA is reduced or even withdrawn.	Qualitative design with interviews.	Parents’ experiences are that PA allows them to fulfill their parental roles.
PubMed	BMC Health Services Research	2019	Personal assistance AND care AND disability	Kristina M. Kokorelias, Monique A. M. Gignac, Gary Naglie and Jill L. Cameron	2	Towards a universal model of family-centered care: a scoping review	To explore existing models of family-centered care to determine the key components of existing models and to identify gaps in the literature.	Scoping review	Healthcare policies and procedures needed that incorporate FCC to create system-level change. Our review moves the field of FCC forward by identifying the universal and illness-specific model components that can inform model development, testing, and implementation.
PubMed	Ann Ist Super Sanitá	2020	Care AND disability	Laura Camoni, Angelo Picardi and Aldina Venerosi	4	A new mode of care. Value and limitations of the person-centered care planning for people with mental disability.	Examines and summarize international research and non-research material to survey the different implementation strategies of personalization in different countries, with a special focus on Italy.	Narrative review	Though decentralization is one of the new modes of care, the central government plays an important role in guiding processes and locating investments and infrastructures suitable to guarantee the quality, equity, and equal opportunities to people with long-term and chronic care needs. In Italy, policies addressed to harmonize welfare rules and opportunities, and to promote social investment and a stable monitoring framework, are urgently needed.
Scopus	Gender Work Organization	2018	Personal assistance AND care AND disability	Cecilie Basberg Neumann and Tonje Gundersen	5	Care parading as service: negotiating recognition and equality in user-controlled personal assistance	To investigate the experience of having a body that is someone else’s area of work as well as the experience of having another person’s body as work focus.	Qualitative design with interviews.	If we instead place the conceptualization of care in a position based on an ethics of responsibility and cooperation (Mol, 2008), the competent sensitivity performed by the good assistant could be based on an interdependent professional ethos for the PAs, as collaborators.
Scopus	Work, Employment, and Society–WES	2022	Personal assistance AND disability	Jane Maddison, Jennni Brooks, Katherine Graham, and Yvonne Birks	3	‘They exist but they don’t exist’: Personal assistants supporting physically disabled people in the workplace	Explores workplace personal assistance as invisible work.	The data analysed here are from a study (during 2016–2017). The design was qualitative, using semi-structured interviews.	Socioculturally, WPAs are subject to layered invisibility, owing to the dual mechanisms of empowerment and workplace ableism operating on the disabled worker. These mechanisms intersect, rendering the WPA role invisible as a tool to empower disabled people, which is in a disregarded zone of the labor of disabled workers.
Scopus	ALTER–European Journal of Disability Research	2019	Personal assistance AND disability	Elisabeth Olin and Anna Dunér	2	Careful assistance? Personal assistance within the family as a hybridization of modern welfare policy and traditional family care.	To examine how different ideological perspectives on Swedish disability policy, are reflected in the experiences of disabled people and their families’ personal assistants.	Qualitative design with interviews.	It became obvious that family assistance could add value to the life situation of both family PAs and assistance users, as in the family as a complementary approach in which both parties were empowered to take control through opportunities of flexible and protected welfare support.
Scopus	Scandinavian Journal of Disability Research	2022	Personal assistance AND disability	Dikmen Bezmez and Tom Porter	5	Disabled Women’s Care experiences in Turkey: Intimacy, dependency, independent living	Analyses the experiences of three disabled women with distinctive care arrangements (paid professional, familial informal, and an eclectic mix).	Case of study (3)	Cultural and political economies of care are key to understanding the experience of support relationships and independent living: the stark deficit of support within the Turkish state, and the deeply gendered structuring of care, interrelate in ways that limit choice and control. Rights-based approaches to welfare—involving direct payments, personal assistance, and an overarching philosophy of IL—have international relevance and the potential to counter-cultural and political barriers to independent living.
Scopus	Disability and Rehabilitation	2022	Personal assistance AND disability	Heléne von Granitz, Karin Sonnander, Ieva Reine, and Ilrika Winblad	1	Do personal assistance activities promote participation in society for persons with disabilities in Sweden? A five-year longitudinal study	To explore whether the personal assistance (PA) activities provided by the Swedish Act concerning Support and Service for Persons with certain functional impairments.	Longitudinal study	The results show that PA activities are used more for medical care and home-based services over the five-year period.
Scopus	Disability and Society	2021	Personal assistance AND disability	Deirdre Nally, Sean S. Moore, and Rosemary Joan Gowran	1	How governments manage personal assistance schemes in response to the United Nations Convention on the Right of Persons with Disabilities (UNCRPD): A Scoping Review	To map and explore current knowledge on how governments internationally have managed PA schemes in response to the UNCRPD.	Scoping review	This paper suggests that we need to have the voice of the PA user to direct the design and delivery of PA schemes.
Scopus	Caring Culture	2019	Personal assistance AND disability	Rubén González-Rodríguez, Carmen Verde-Diego, y Violeta Pérez-Lahoz	3	Personal assistance as a new citizen right: a perspective from health professionals’ outlook	Knowing about the figure of the personal assistant for people with disabilities and describing the importance of care and professionalized accompaniment vis-à-vis family care.	A qualitative design with interviews and official database reviews.	The nurses interviewed considered that personal assistance functions should be focused on clear professionalization for its specific characteristics.
Scopus	International Journal of Care and Caring	2018	Personal assistance AND disability	Tom Shakespeare, Andrea Stöckl, and Tom Porter	3	Metaphors to work by: the meaning of personal assistance in England	To explore the meaning of personal assistance in England through metaphors.	Qualitative design with interviews.	When personal assistants come from a different culture there may be additional confusion about the different metaphors the employer and worker may be drawing upon. Conflict can emerge from ‘crossing boundaries’, which may be implicit and hidden.
Scopus	Nordic Social Work Research	2022	Personal assistance AND disability	Ann-Sofie Bergman, Ulla Melin Emilsson, and Ulrika J. Berggren	2	Persons with certain functional impairments apply for parenting support: a study of personal assistance assessments in Sweden	Explore what kind of support parents who have functional impairments and apply for personal assistance may need when they have underage children.	Based on qualitative and quantitative document analyses.	The general perception in Sweden that the welfare state protects children may contribute to a lack of recognition. There is a risk that children may become young careers in Sweden due to the construction of the welfare system that is individual-based and lacking in a child perspective and family perspective in the distribution of welfare rights.
Scopus	European Journal of Social Work	2021	Personal assistance AND disability	Ulrika J. Berggren, Ulla Melin Emilsson, and Ann-Sofie Bergman	3	Strategies of austerity used in needs assessments for personal assistance—changing Swedish social policy for a person with disabilities.	Examines the strategies used by public officials in implementing austerity measures in needs assessment for personal assistance in Sweden.	Based on a document study with N: 100 records of needs assessment for PA for a person with serious functional disabilities.	The social policy values of fifty years, emphasizing the right to equal participation in society, are traded for economic austerity goals.
WoS	Scandinavian Journal of Disability Research	2018	Personal assistance AND care AND disability	Line Jenhaug and Ole Petter Askheim	2	Empowering Parents as Co-producers: Personal assistance for families with disabled children	To what extent co-producing PA with the municipality empowers the parents as family managers.	Qualitative design with interviews.	PA makes parents feel more empowered and improves their control and coping. It also gives their children the benefit of both parental care and increasing independence. However, in the decision-making process of granting and following up PA, the parents also experience that they are not regarded as equal co-producers by the municipal services.
WoS	Disability and Society	2020	Personal assistance AND disability	Teodor Mladenov	5	What is good personal assistance made of? Results of a European survey	Looks at the results of a survey on personal assistance for disabled people in Europe.	The survey presented in this article was constructed by elaborating a series of statements describing various typical characteristics of PA based on a literature review that included academic papers and civil society reports.	The greatest enabler of choice and control in a PA scheme is the opportunity to choose one’s personal assistant. As far as the greatest barriers are concerned, they include restrictions over the ‘who’, the ‘where’, the ‘what’, and the ‘when’ of PA. Of particular concern are cuts and/or funding restrictions imposed on PA.
WoS	Social & Cultural Geography	2022	Care AND disability	Carey-Ann Morrison	4	A personal geography of care and disability	This research focuses on the bodies, feelings, spaces, and places of care and disability.	Autobiography	This article concludes that any attempt to understand the care needs to consider the everyday realities of caregivers and the paradoxical embodied and emotional spaces they occupy.
WoS	Disability & Society	2022	Care AND disability	Shixin Huang	4 y 5	Activating disability care: the formation of collective disability care networks in China’s COVID-19 outbreak	Examines the evolution and development of two collective care networks formed by and for people with disabilities and their family members during the COVID-19 outbreak in China.	Qualitative content analysis.	Care (and guardianship) in this context refers not merely to a political identity (although increasingly so with a new generation of disability rights activists), but basic needs of everyday survival. Public ethics of care in this context thus also implied a reconstitution of disability politics towards a fairer social distribution of care responsibility and the recognition of people with disabilities as active citizens and political agents.
WoS	International Journal of Care and Caring	2020	Care AND disability	Poland Lai	5	Care, rights, and disability	Makes a cautious case against unequivocal acceptance of the rights paradigm for carers.	Theorist analysis	In a caring relationship, the interests and identities of all parties are intermingled, and it is impossible to consider the welfare or rights of any one party in isolation. Examples in the long-term care sector (nursing homes) in Ontario, Canada, will be used to illustrate situations where the use of rights can be respectful for all parties in a caring relationship.
Scielo	Feminist Studies Journal	2022	Care AND disability	Marivete Gesser, Ilze Zirbel, and Karla García Luiz	5	Cuidado na dependência complexa de pessoas com deficiência: uma questão de justiça	Problematizar o cuidado de pessoas com deficiência que experienciam a dependência complexa e defendê-lo como uma questão de justiça.	Revisão teórica	Nossas análises foram pautadas no diálogo entre os estudos da deficiência e uma ética político- feminista do cuidado. Elas indicaram que não é possível garantir os direitos das pessoas com deficiência que experienciam a dependência complexa sem romper com o capacitismo e as políticas familistas, reiteradas por governos neoliberais.

Source: Table created by authors.

## 3. Results and Discussion

### 3.1. Personal Assistance Dimension: A Tool for People with Disabilities to Achieve Autonomy

Personal assistance arose within the framework of the Independent Living Movement (ILM), which defends the idea of inclusion for people with disabilities as legal subjects. The driving ideas guiding ILM philosophy are deinstitutionalization, de-medicalization, and de-professionalization [13].

In this regard, the figure of the personal assistant is not considered to be a caregiver, but rather a necessary figure, with a job contract and payment, in order for people with disabilities to achieve an independent life. According to Rafael de Asís [1], personal assistance takes shape as part of the right to be included in society.

Along these lines, and paraphrasing Prieto, de la Rosa & Izquieta [14] personal assistance is a “service” allowing people with disabilities to enjoy their autonomy while they carry out their life projects. In line with this idea, Iáñez, Aranda & García [15] agree that personal assistance is a “service” given that it involves an economic impact for the States which provide it. In this sense, they mention that the personal assistance project has generated potential savings for its beneficiaries compared to if they paid for it in the private market. They thus emphasize the need for public funding in order for all people with disabilities, regardless of where they live, can have the same opportunities to achieve independent living.

There is little scientific production in Latin American countries regarding personal assistance, with the only article from this search referring to the study by Minoletti et al. [16] from Chile about the design of a brief personal assistance model to be carried out by community agents with professional supervision, which showed certain weaknesses linked to the lack of regional-level studies about the topic and the Latin American cultural understanding about informal support from families and community agents.

However, within the European reality, von Granitz et al. [17] stated that personal assistance is incorporated into Swedish law about support and service for people with functional deficiencies (LSS), which to a certain extent is an expression of social commitment from national public policy. Despite this, the authors indicate that personal assistance has incorporated more health and care activities instead of providing activities for citizen participation. They thus raise concerns that the rise in personal assistance hours does not always lead to more participation by people with disabilities in society or outside the home.

For their part, Nally, Moore & Gowran [18] consider the emergency of personal assistance in various countries and how it has had various traits attributed to it in different places. The authors emphasize two main attention schemes. They classify them as either provider-led models, where the provider has the most options and control over the service; and the user-led model, which fits the directives established by the ILM.

### 3.2. Dimension: The Familiarization of Care Versus Personal Assistance of People with Disabilities

The familiarization of care for people with disabilities has been a widely approached and questioned topic, especially in the scene which is more critical of the disability and independent living social movement.

Along these lines, one concept emerging from the discussion is “de-familization”, defined by Ruther Lister [19] as “the degree to which adults can maintain an accepted social position, independent of family relations” [19] (p. 57). According to this, the study by Rauch, Olin & Dunér [20] indicates the need for States to incorporate personal assistance within their social rights. They also emphasize the importance of personal assistance as a mechanism to avoid mutual dependency between people with disabilities and their families.

Kokorelias et al. [21] indicated that the care given by family, rather than being eliminated from care dynamics, should be transformed in order to help strengthen its quality via policies and programs which help uphold caregivers’ roles. This will help reduce the negative consequences of at-home care.

In sync with the previous points, Olin & Dunér [22] indicated that personal assistance provided within the family can be seen as a hybrid between publicly regulated work and paid work carried out within the family sphere. They added that family assistance can have advantages such as providing personalized services, but they also indicate the existence of disadvantages mainly associated with undesired dependency and the risk of leading to a family-level “break”.

Some other studies considered the complex interaction rising from the parental relationship between parents with disabilities and caring for their children. Berggren & Bergman [23] and Bergman, Emilsson & Berggren [24] mentioned that social discourses about disability do not consider parenthood as a role for adults with disabilities. From a parenting perspective, there is a questioning of the parents’ identity since their work as legitimate caregivers are not recognized. Some parents thus show a certain fear in requesting personal assistance due to being seen as unfit for care, or in more extreme cases, losing custody of their children. However, testimonies from other parents who did receive personal assistance services mentioned feeling supported in their efforts, as it helped them fulfill their parental role more efficiently.

Another study analyzing personal assistance among families with disabled children [25] indicated that PA allowed parents to feel more empowered in their role, improving control and the ability to face matters regarding their children. In this sense, the authors affirm that PA is a direct aid for parents in childcare, with the parents also becoming users of the service.

### 3.3. Dimension: Contractual Vulnerability of Personal Care and Assistance Practices

One rarely approached angle in the articles we found was the contractual situation faced by personal assistance and/or those who perform these functions.

Christoph Tschanz [26] described the long trajectory of social protection represented by personal assistance in countries including Switzerland, Sweden, Germany, and the UK. However, López-Pérez & Álvarez-Nieto [27] and González-Rodríguez, Verde-Diego & Pérez-Lahoz [28] indicated that in countries such as Spain, the situation is very different. In contractual terms, they describe the presence of a professional contract, which must be regularized in order to proceed with personal assistance functions and add that this work figure is a key cog in the machinery for guiding independent life via a professional who performs the functions agreed on in the contract in exchange for payment.

However, Berggren, Emilsson & Bergman [29] raised the alarm about the economic precariousness faced by personal assistants. Specifically, they noted that austerity policies regarding social rights are impeding the injection of more and better resources for personal assistance.

Another variable mentioned by López-Pérez & Álvarez-Nieto [27] is the profile behind the figure of the personal assistant, as these are mainly women, immigrants, and/or students. This is related to the low pay they receive (1000 €) and their workload of 38.5 h/week.

Shakespeare, Stöckl & Porter [30] added that personal assistance has given more freedom to people with disabilities, allowing them to actively participate in daily life. However, this is possible with adequate funding and when people do not have to live in institutions or be left as charges of family members. These elements require people with disabilities to become employers to administer budgets, hire people, and manage personal assistants’ salaries. In this sense, one positive aspect highlighted by the authors is flexible working conditions, which can be attractive for people seeking informal or part-time employment. However, the negative aspect is related to work conditions, insecurity, and lack of regularization, as well as the close connection seen in most cases between the PA and people with disabilities, a matter which creates confusion between their duties and their feelings for their employers.

Similarly, Porter, Shakespeare & Stöckl [31] add the moral and emotional dilemmas which PAs have mentioned about their intent to define the personal and professional parameters of their relations. The “invisible” image becomes virtuous in order to go unnoticed as a characteristic of quality care. However, the authors mentioned that this invisibility denies voice and recognition to the workers (PAs) who are often young, underpaid women. The authors conclude that the liberation of people with disabilities involves making progress and advances by depriving others of their rights.

Similar to this perspective, Maddison et al. [32] add that for people with disabilities, participation in paid work has been possible thanks to the figure of the PA. In this sense, the few studies on the matter have mainly approached their role in the home, but few studies have analyzed the role of PA in disabled persons’ workplaces, which for some authors is understood as an “invisible job”, a conceptualization arising from the epistemic trajectory of unpaid housework carried out by women. Its application in this case implies the invisibility of personal assistants behind the dual mechanisms of empowerment and work capacity operating in the disabled worker. These mechanisms generate the “invisibilization” of the personal assistant when they cross over.

When considering ideal traits for personal assistants, Ruiz, Rodríguez & Izuzquiza [33] mentioned the existence of basic skills to facilitate interactions between people with disabilities and their PA. These are the ability to establish a non-directive communication style with the user; active listening skills; non-verbal communication control; respect for the time that users require to express and share their opinions; teamwork and support network coordination skills; and respect for users’ personalities.

In this regard, Silvia Federici [34] mentions that one fundamental care problem is the lack of recognition for it as a paid job activity, an important part of what she called “the wages of the patriarchy”. Along these lines, she states that there is a historic debt to the work carried out by women to uphold economic organization, where a gendered division of labor places the domestic sphere as private and grants no social recognition to caring for people with disabilities.

### 3.4. Ethical Dimension of Care towards People with Disabilities

Caring for people with disabilities has been frequently interpellated by the Independent Living Movement since it challenges the autonomy and independence of people with disabilities. In his study, Diego Carmona Gallego [35] considers the notion of autonomy and how its ontological basis includes various analysis perspectives. In this sense, he indicates the mechanicist perspective centered on individualism and self-reliance, and the care ethic perspective centered on an interdependent dimension of life. In the face of both perspectives, the author proposes relational autonomy with contributions from feminist philosophy, which considers autonomy as emerging from a singularity related to connected narratives.

From the perspective of the geography of care, Carey-Ann Morrison [36] indicated that care is a complex set of experiences, physical practices, and policies that connect with people and spaces. In this sense, the geography of care allows for a much deeper comprehension of the life trajectories of people with disabilities and their caregivers, as well as the meaning of their spaces, since care is understood as “emotional, incarnated and constantly arising via commitment with material and discursive spaces and places” [36] (p. 1045). Despite this, the author recognizes that care has been a widely criticized concept among disability activists and academics due to its links with incapacity and dependency. However, she agrees with the statements from other authors who promote the notion of an “ethics of disability care” which implies interdependency between disabled people and their caregivers.

The understanding of interdependency is habitually found within articles discussing care ethics. Shixin Huang [37] develops this idea by highlighting the premise of collective and social care for people with disabilities during the COVID-19 Pandemic. The author mentions that support networks provided attention and care both for people with disabilities and their environment, adding that interdependency and social responsibility fit within the new logic for understanding care: centered on people, but also the community.

Other conceptual proposals from the healthcare sector are observed in the debate, such as that developed by Camoni, Picardi & Venerosi [38]. The political and socio-sanitary model termed a “welfare community” is related to the principles of equity, solidarity, and participation. According to the authors, under this model, the users are protagonists of their health project and the resources assigned, which also grants them both autonomy and independence.

### 3.5. Dimension: Tension between the Independent Living Movement and the Feminist Movement on Personal Assistance and Care

Personal assistance has been positioned as an ideal care model, since it grants autonomy and independence to people with disabilities, although it has not been free of controversies and challenges. Along these lines, Neumann & Gundersen [8] describe the tension between feminist demands regarding care and PA from the Independent Living Movement. Since the 1960s, ILM participants with disabilities have fought for more autonomy, a similar outlook to that of the feminist movement in its fight for equality and recognition. However, for people with disabilities care connotes oppression, vulnerability, and devaluation, while for feminist theorists, care implies a place from which to acquire more visibility and political commitment.

Bezmez & Porter [39] agree with this, although they warn that this discussion, which is often theoretical, does not address the reality of other geopolitical sectors, and is more centered on the experiences of countries such as the UK, USA, or Norway. The authors thus conclude that recognizing other geographical experiences will contribute to a broader comprehension of people with disabilities and care theories.

Another interesting point regarding this tension comes from Teodor Mladenov [40]. The author mentions the importance of the emotional aspect in the relationship between PA users and the PAs themselves. Along these lines, feminist theories on care ethics have expressed their concern for the functioning and job conditions that PAs have faced, as they are mostly women with highly vulnerable life trajectories. Similarly, they have presented the mercantilizing component of assistance, destabilizing its communitarian and collective understanding. The author mentions cases where PA work has led to labor exploitation among women, particularly immigrants.

From a legal framework, Antonio Pau [41] mentions that the principles of “equality” and “care” for disability contain conceptual differences which can interfere with the conception of assistance and care. When considering the equality principle, he mentions this promotes equal treatment for all people regardless of their condition, unlike the care principle which implies understanding people as unequal, since it is rooted in dependence and vulnerability. On this path, the feminist ethic of care and disability raises an interesting debate beyond the conceptual. However, Shixin Huang [37] adds that in the debate there are important meeting points, indicating that there are “alternative visions to the dependency of vulnerable bodies and minds and [feminist care ethics] proposes ideas such as relational citizenship, connection-based equality, and democracy through solidarity, which recognize and value interdependent relationships” [37] (p. 4). The author adds that the preceding must also include a public ethics of care which recognizes its social responsibility.

For their part, Gesser, Zirbel & Garcia Luiz [42] and Poland Lai [43] state that care from women (without disabilities) as caregivers do not visibilize the needs of women who receive care, i.e., those with disabilities. In this sense, Lai [43] mentions that many women with disabilities who practice care are not recognized, even by state policies. The author proposes the focus from Jonathan Herring about care relations by stating that “instead of promoting a paradigm of independent rights for caregivers, we need to center on the rights of support or other legal interventions which promote the interests of everyone involved in a care relationship” [43] (p. 601). Finally, Asún Pie [44] states that demonizing care invisibilizes the unfair social situations where they occur.

## 4. Limitations of the Study

Personal assistance has been worked mainly in countries of the global north. This makes analysis situated from Latin America and the Caribbean difficult. In this sense, future research is expected to address personal care and assistance from an intercultural and decolonial perspective.

## 5. Conclusions

The findings of this review allowed us to discover and analyze scientific evidence about approaches to personal assistance and care for people with disabilities, which show that there is an incipient debate about its various relations and complexities. We see that there is a demand and recognition of various historical needs particular to two important movements which are still active in their identitarian social struggles, albeit with little articulation.

This situation gives rise to politico-epistemic tension at the moment of choosing, exercising, and understanding PA and/or care, a scenario that is often ultimately approached from a binary perspective. This dilemma manifests specific interests in relational, ethical, political, and cultural situations.

It is thus necessary to move towards a collective, situated, and public comprehension, which opens the possibility of thinking about interdependency and autonomy for people with disabilities and their bodies in an emancipated and solidarity-based way, promoting a sustainable, gendered, intergenerational and strategic vision which provides dignity to various life positions and projects.

## Figures and Tables

**Table 1:** Articles found by database and Boolean combinations.

Databases	“Personal Assistance AND Disability”	“Personal Assistance AND Care AND Disability”	“Care AND Disability”	“Asistencia Personal AND Discapacidad”	“Asistencia Personal AND Cuidados AND Discapacidad”	“Cuidados AND Discapacidad”
Scielo	12	0	120	2	2	85
Dialnet	41	19	1135	94	39	568
PubMed	3923	680	95,951	0	0	0
Scopus	977	572	109,166	0	0	11
Web of Science	618	314	57,605	0	0	0
Total	271,934					

Source: authors.

## Data Availability

All information is in the article.
